# Supplementary figures and images for: WRN and WRNIP1 ATPases impose high fidelity on translesion synthesis by Y-family DNA polymerases
Source: eLife. 2025 Sep 3;14:RP106934. doi: 10.7554/eLife.106934 (PMC12408069; doi:10.7554/eLife.106934)

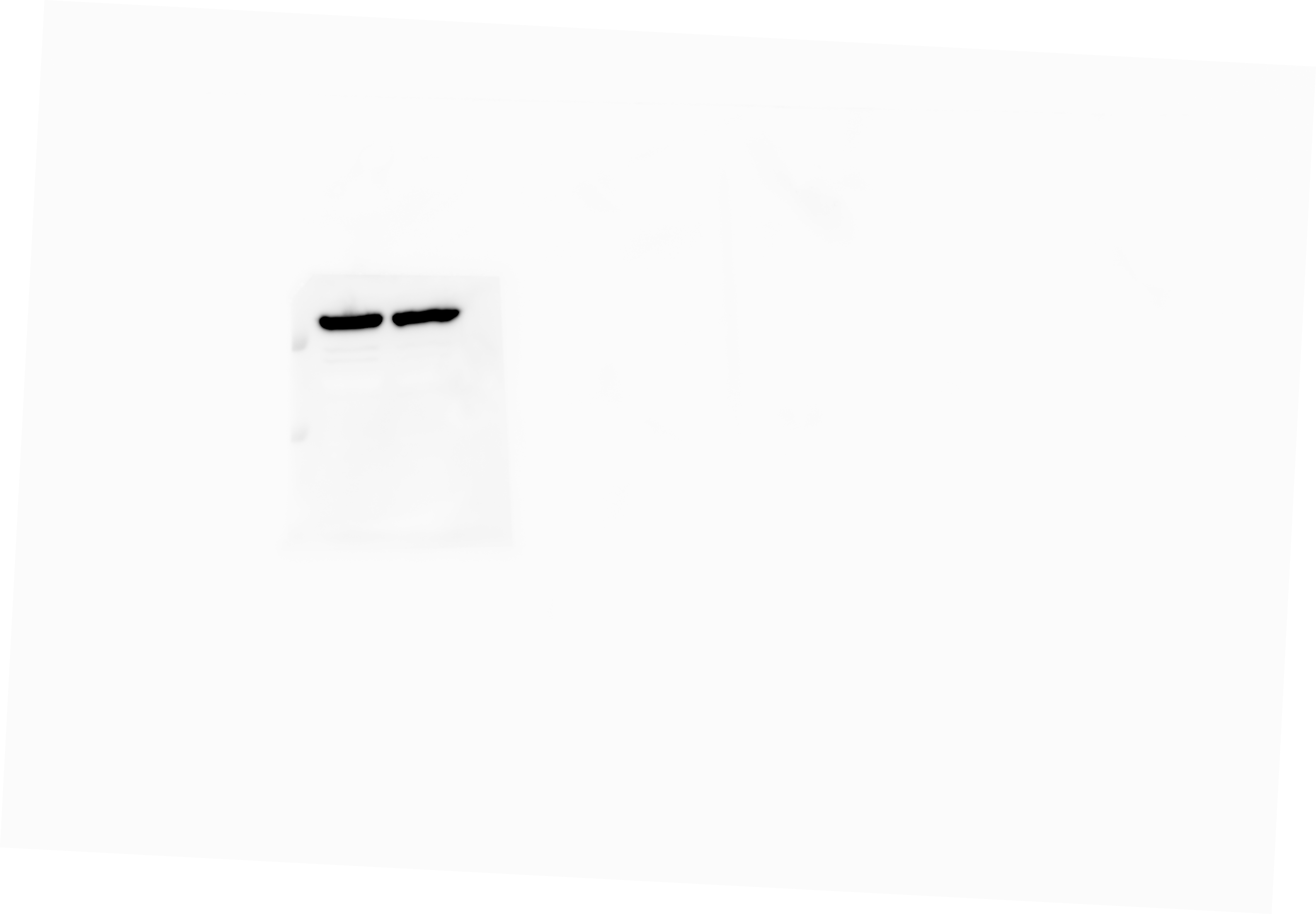

Supplement: Figure 1—figure supplement 1—source data 1. [file elife-106934-fig1-figsupp1-data1.zip › Figure 1 - figure supplement 1 - source data 1/Fig S1A BBMEF b-tubulin ab.tif]

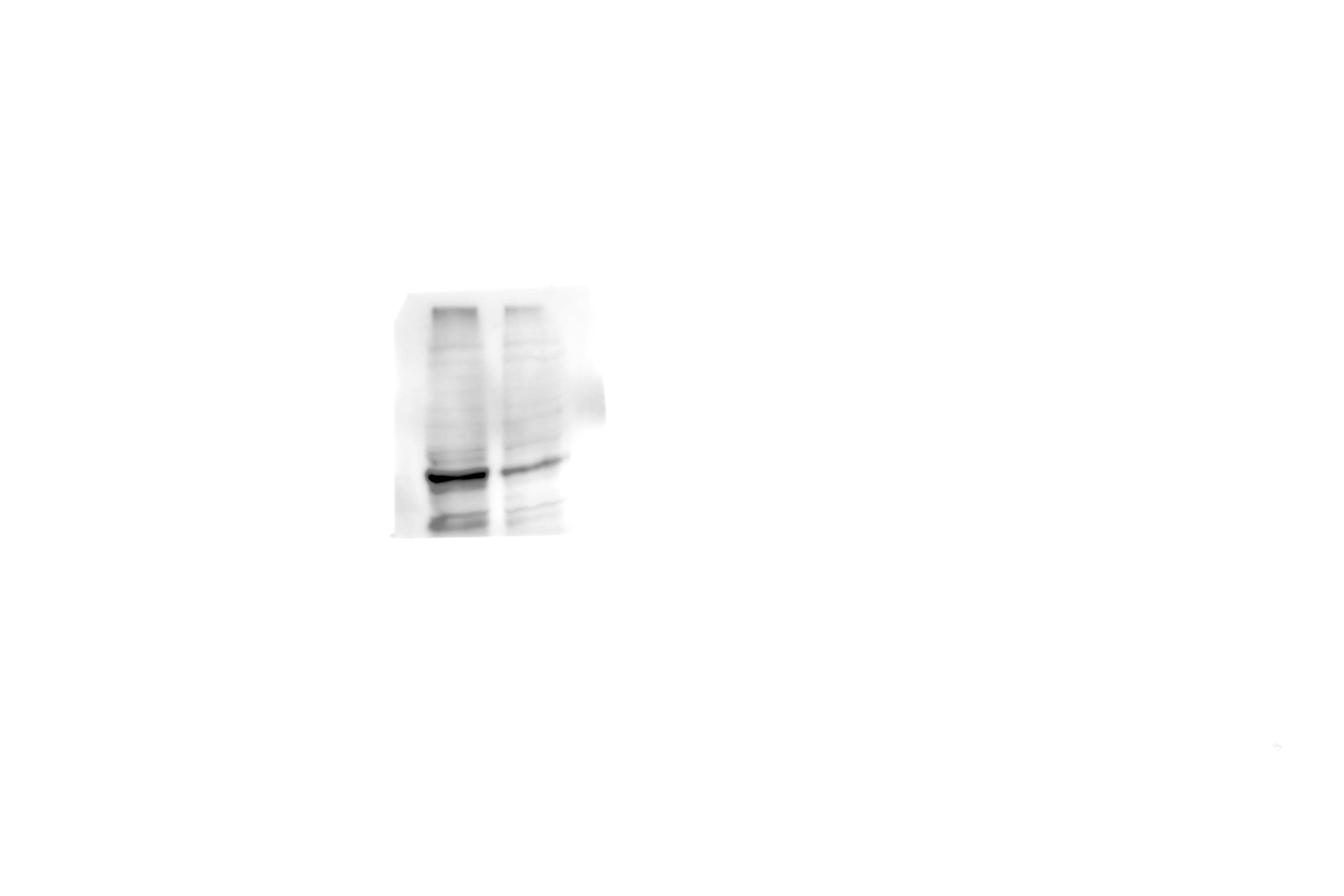

Supplement: Figure 1—figure supplement 1—source data 1. [file elife-106934-fig1-figsupp1-data1.zip › Figure 1 - figure supplement 1 - source data 1/Fig S1A BBMEF WRNIP1 ab.tif]

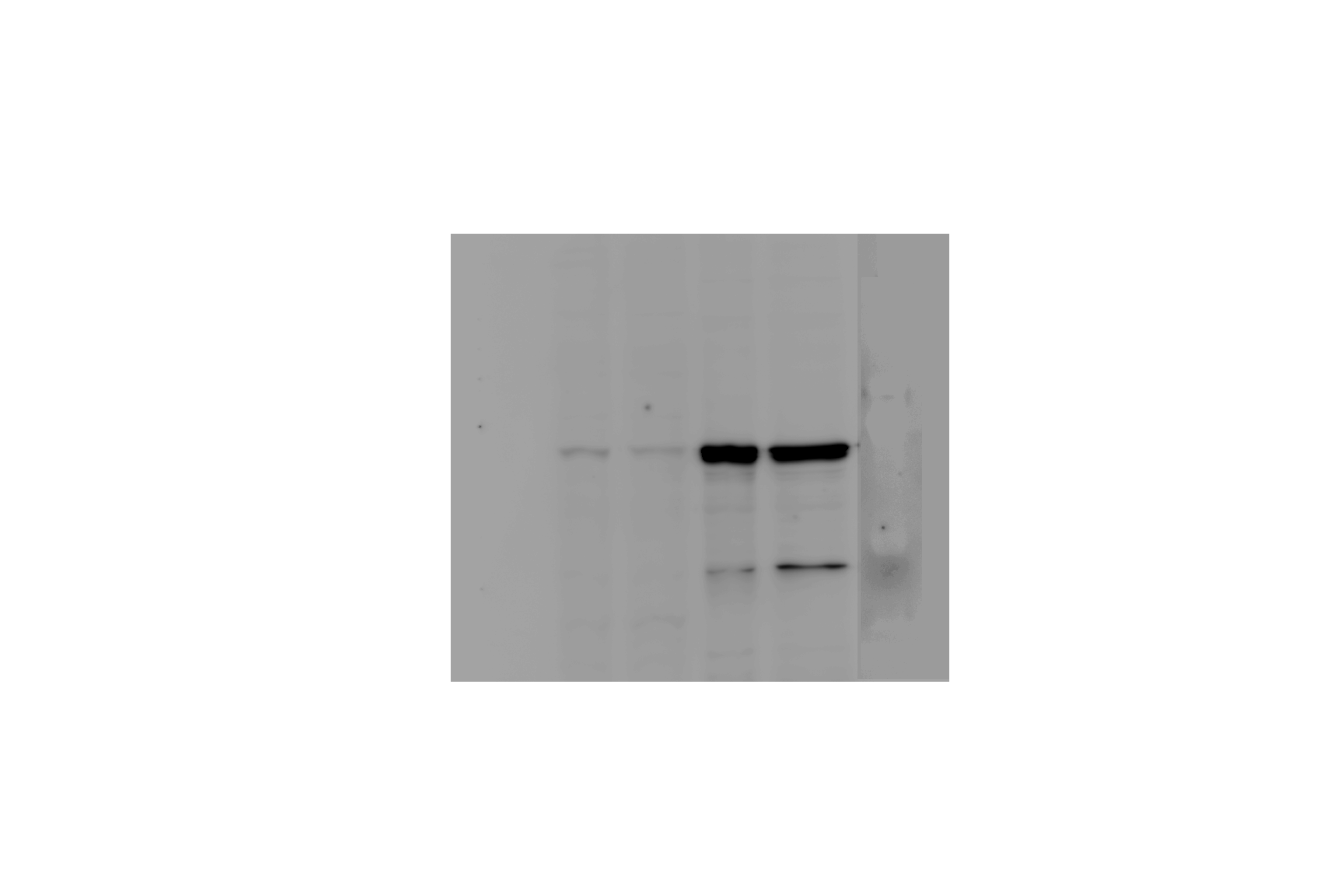

Supplement: Figure 1—figure supplement 1—source data 1. [file elife-106934-fig1-figsupp1-data1.zip › Figure 1 - figure supplement 1 - source data 1/Fig S1A HF LaminB1 ab.tif]

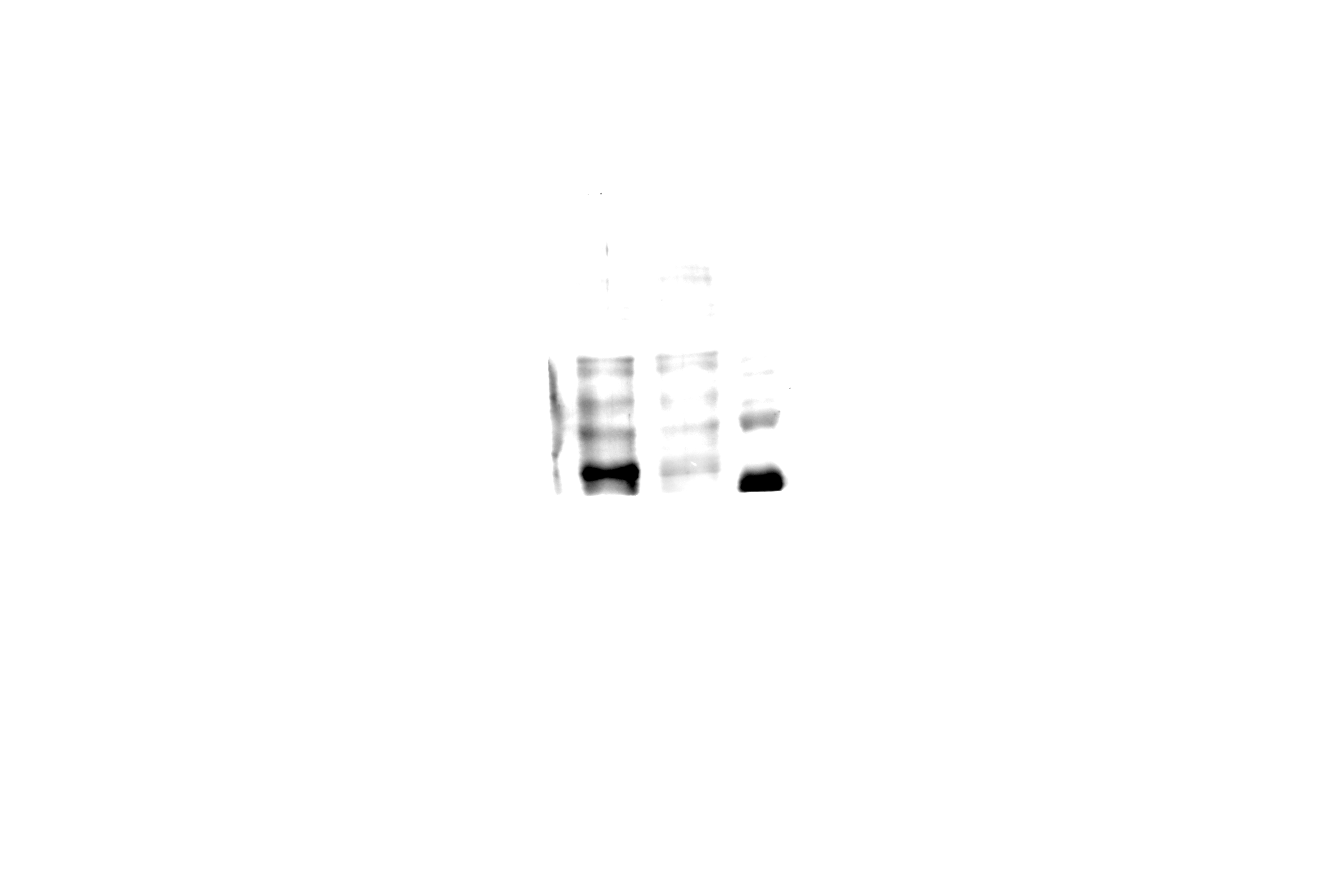

Supplement: Figure 1—figure supplement 1—source data 1. [file elife-106934-fig1-figsupp1-data1.zip › Figure 1 - figure supplement 1 - source data 1/Fig S1A HF WRNIP1 ab.tif]

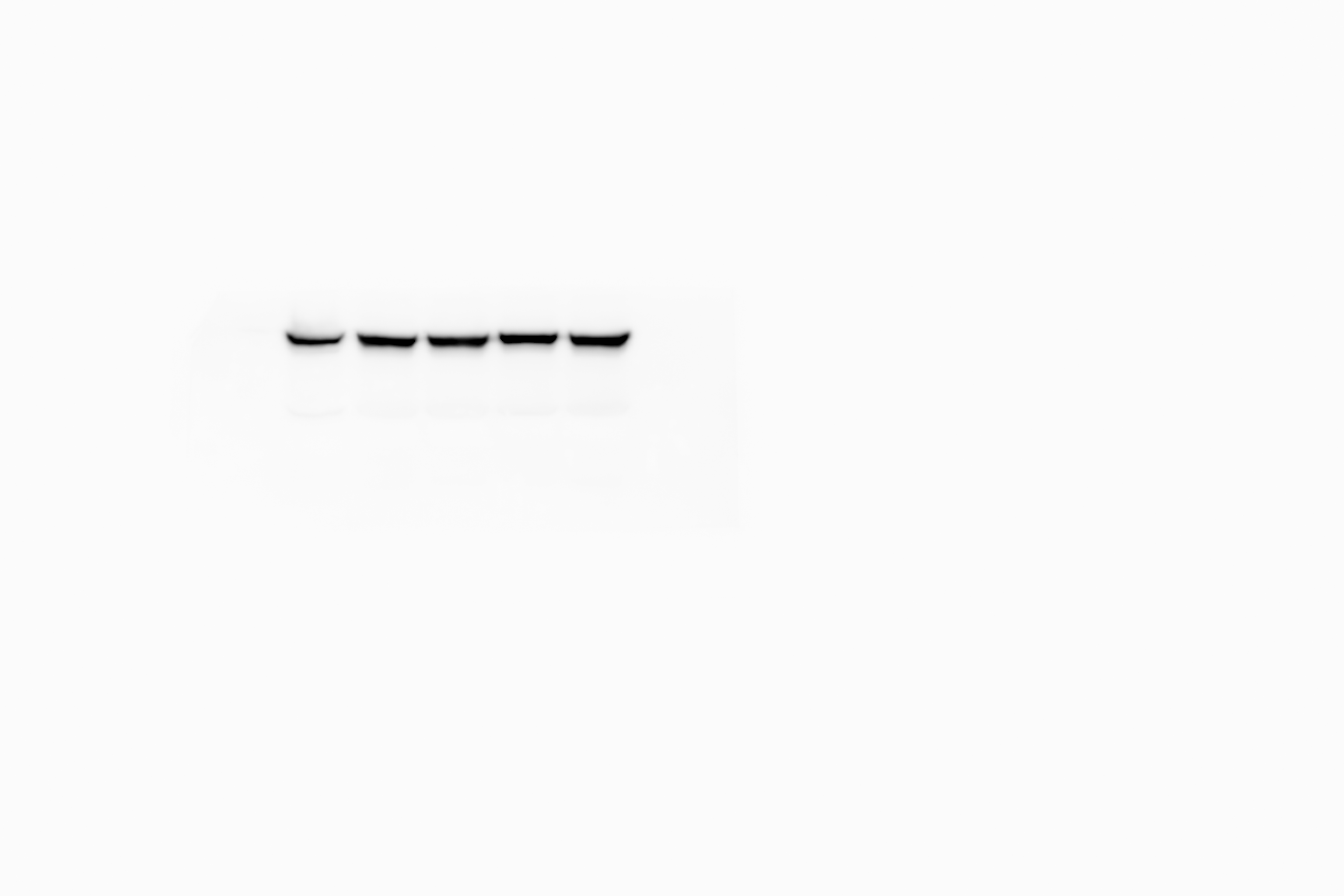

Supplement: Figure 1—figure supplement 1—source data 1. [file elife-106934-fig1-figsupp1-data1.zip › Figure 1 - figure supplement 1 - source data 1/Fig S1B BBMEF LaminB1 ab.tif]

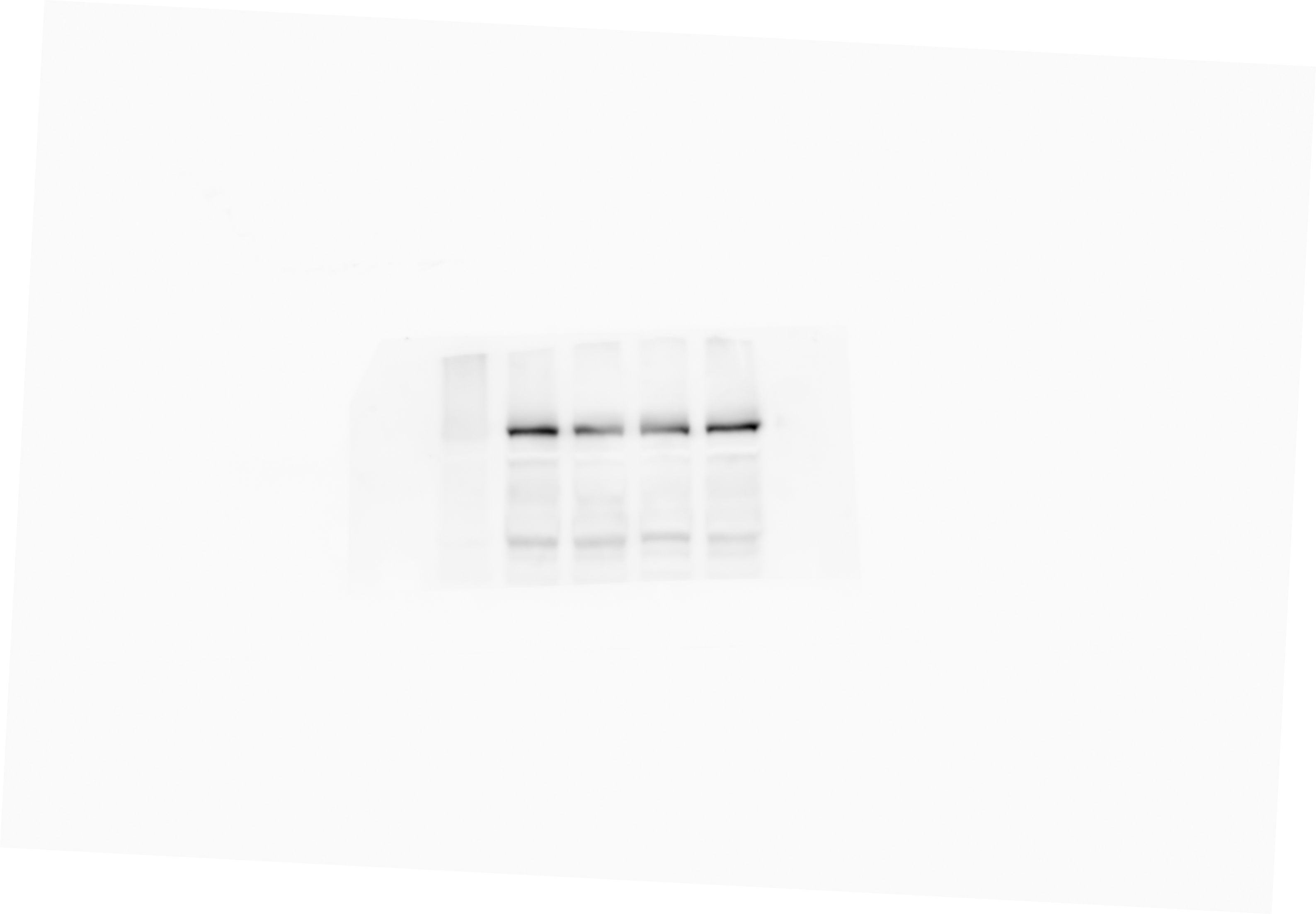

Supplement: Figure 1—figure supplement 1—source data 1. [file elife-106934-fig1-figsupp1-data1.zip › Figure 1 - figure supplement 1 - source data 1/Fig S1B BBMEF myc ab.tif]

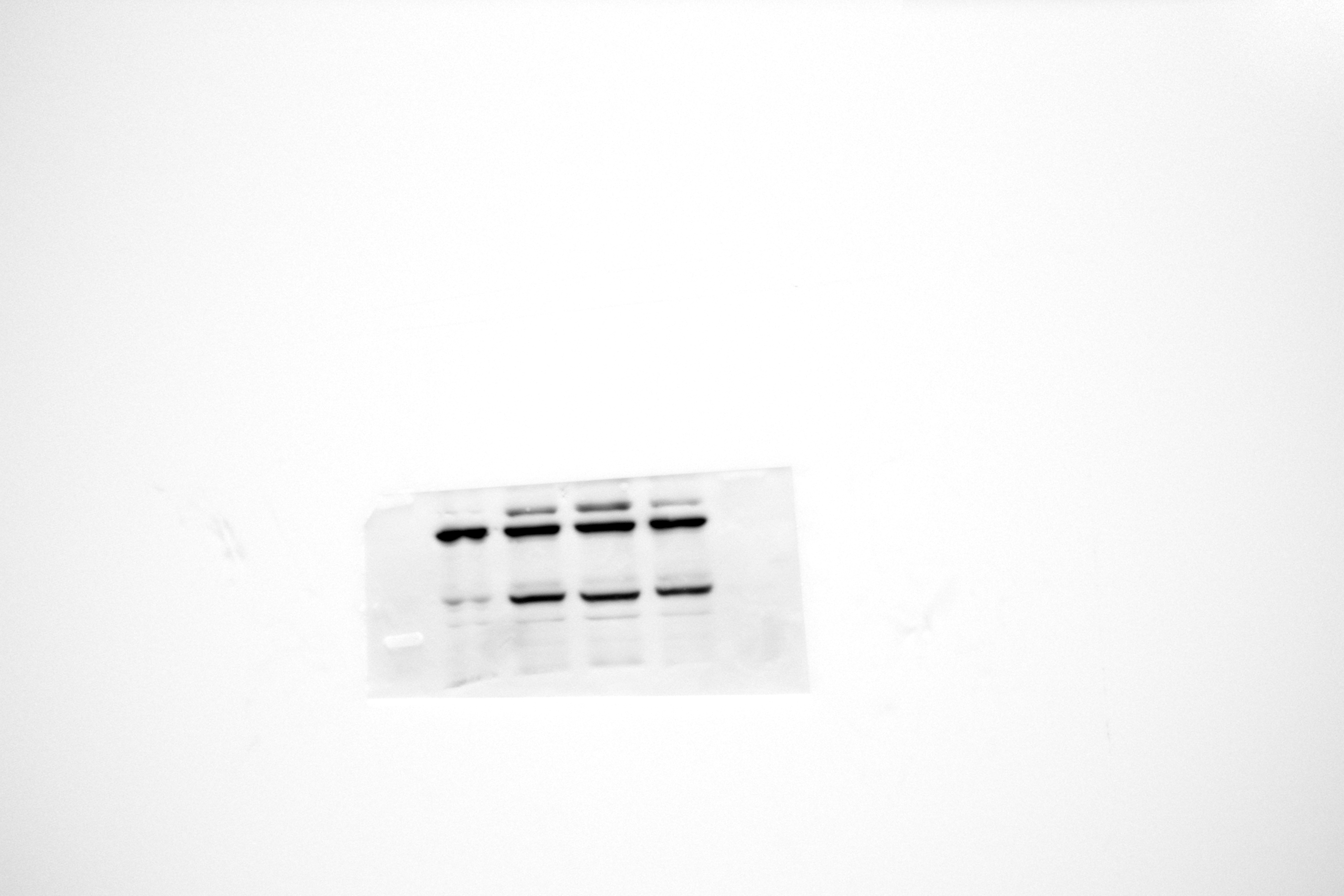

Supplement: Figure 1—figure supplement 1—source data 1. [file elife-106934-fig1-figsupp1-data1.zip › Figure 1 - figure supplement 1 - source data 1/Fig S1B WRN HF LaminB1 ab.tif]

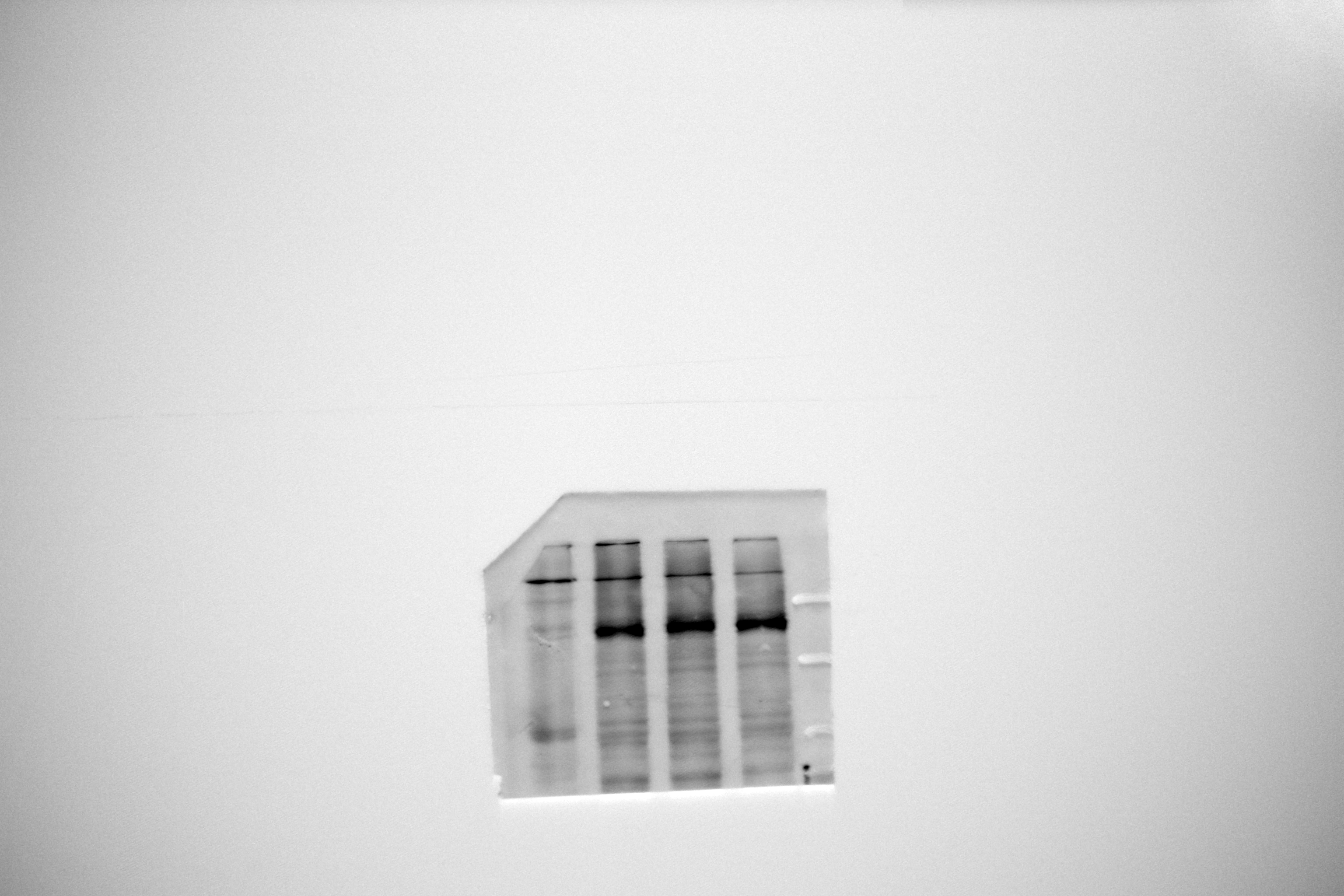

Supplement: Figure 1—figure supplement 1—source data 1. [file elife-106934-fig1-figsupp1-data1.zip › Figure 1 - figure supplement 1 - source data 1/Fig S1B WRN HF myc ab.tif]

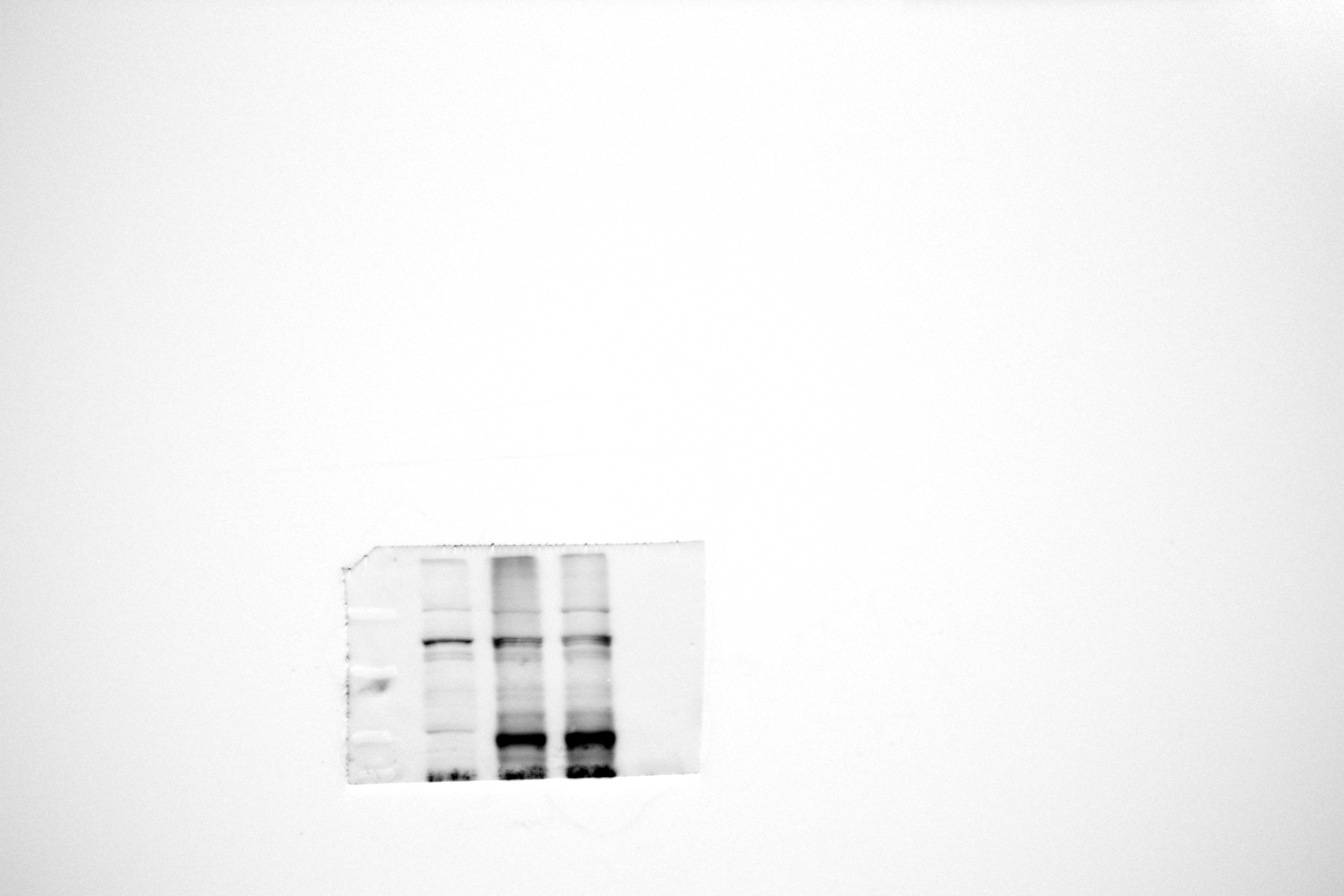

Supplement: Figure 1—figure supplement 1—source data 1. [file elife-106934-fig1-figsupp1-data1.zip › Figure 1 - figure supplement 1 - source data 1/Fig S1C BBMEF Flag ab.tif]

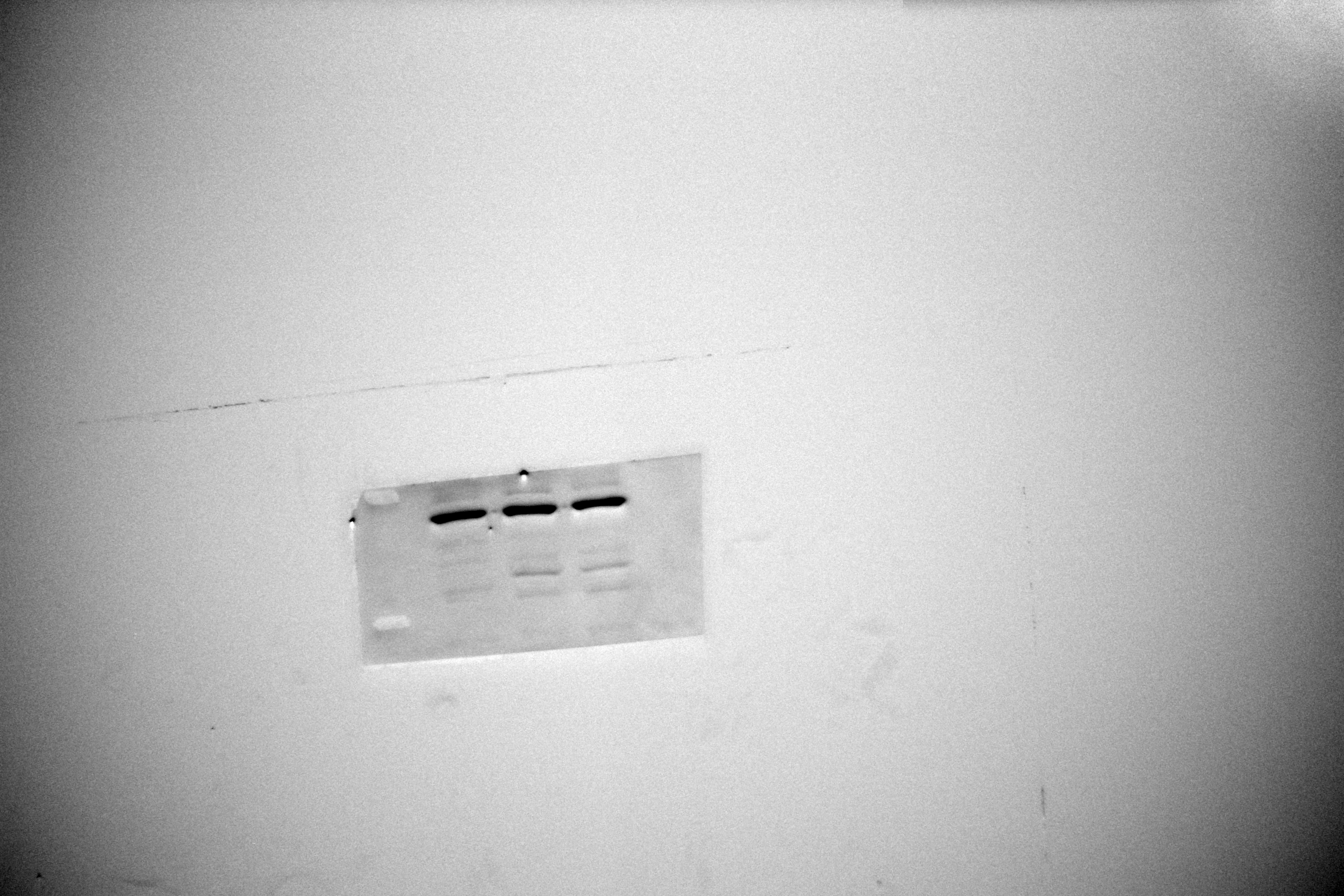

Supplement: Figure 1—figure supplement 1—source data 1. [file elife-106934-fig1-figsupp1-data1.zip › Figure 1 - figure supplement 1 - source data 1/Fig S1C BBMEF LaminB1 ab.tif]

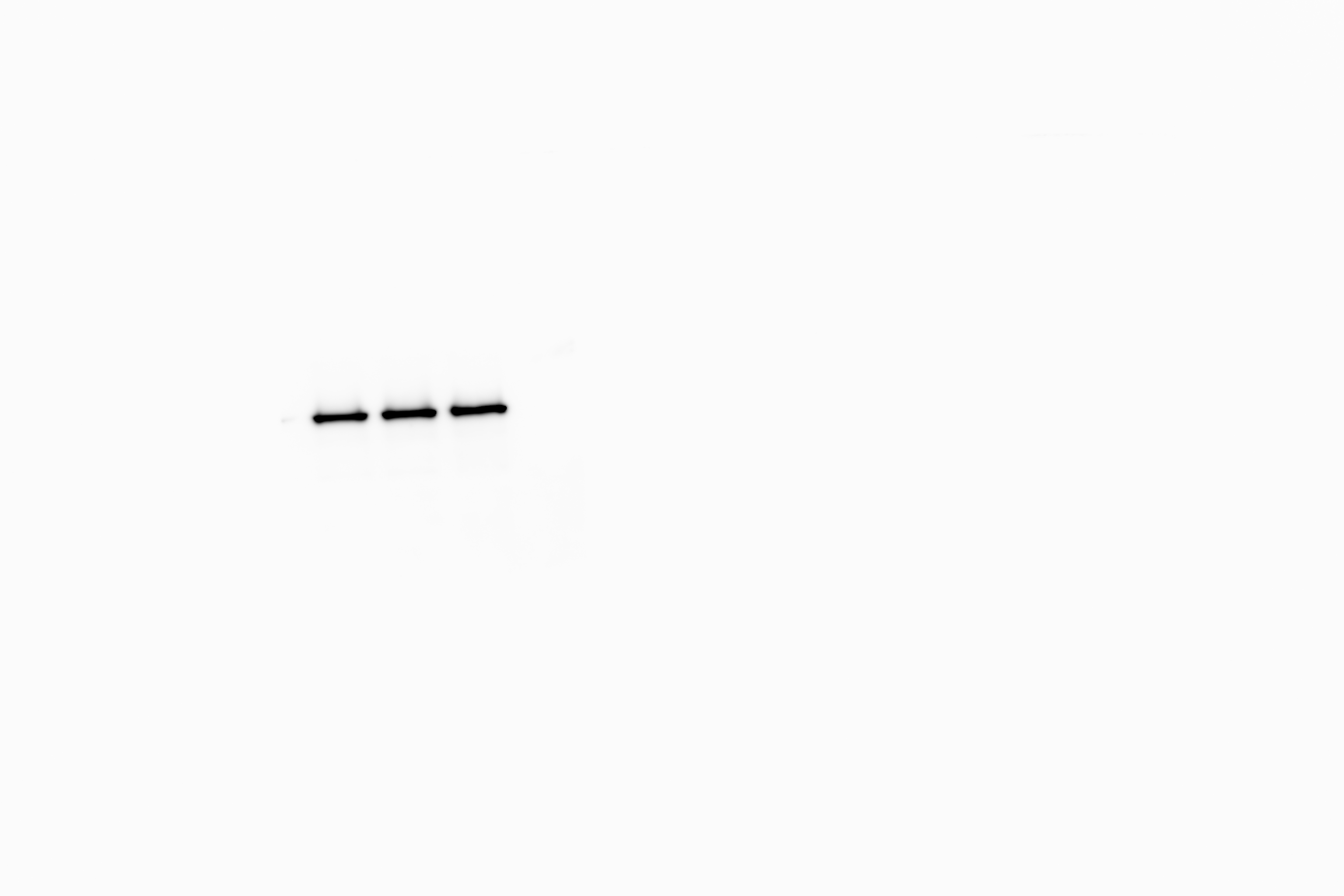

Supplement: Figure 1—figure supplement 1—source data 1. [file elife-106934-fig1-figsupp1-data1.zip › Figure 1 - figure supplement 1 - source data 1/Fig S1C HF b-tubulin ab.tif]

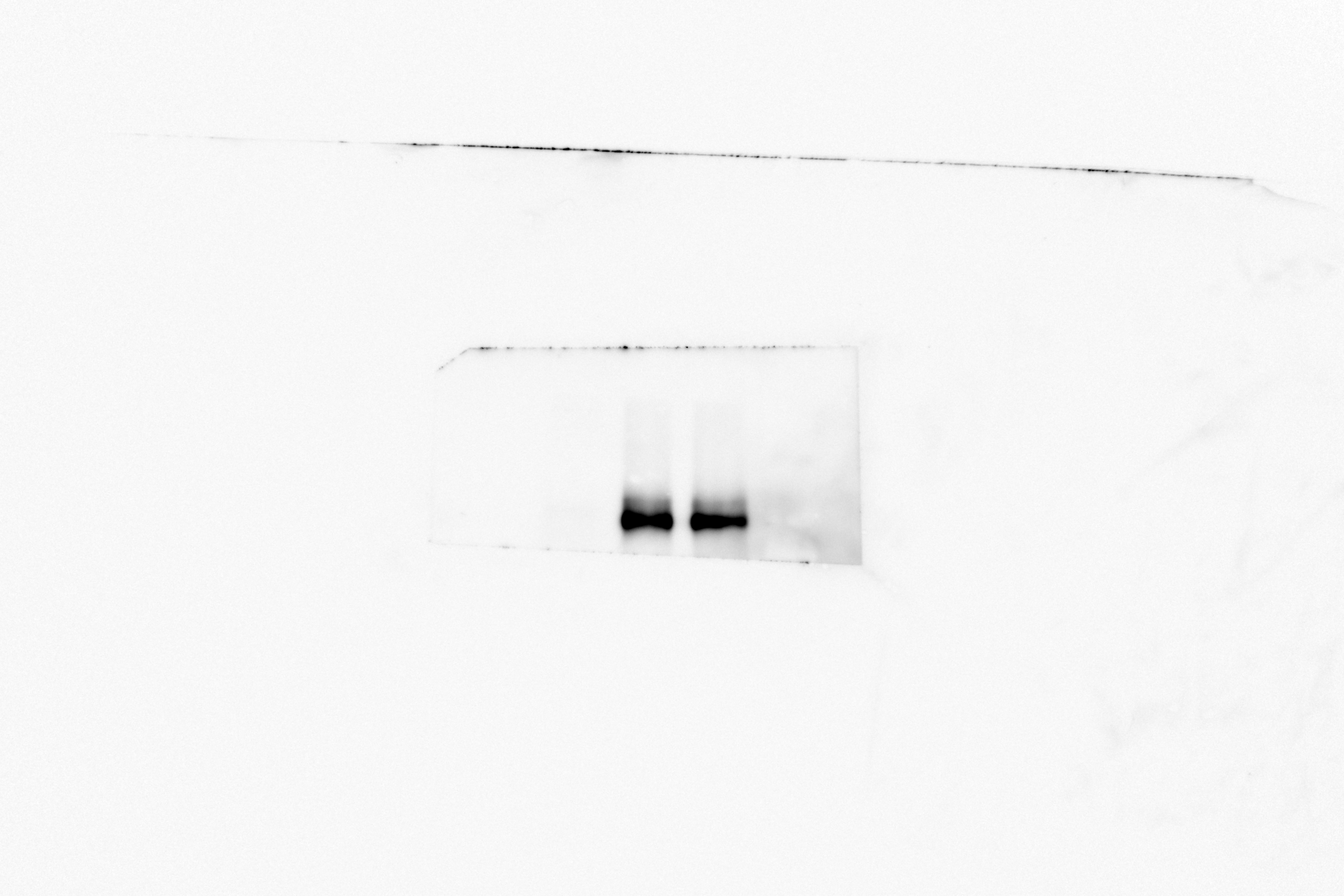

Supplement: Figure 1—figure supplement 1—source data 1. [file elife-106934-fig1-figsupp1-data1.zip › Figure 1 - figure supplement 1 - source data 1/Fig S1C HF Flag ab.tif]

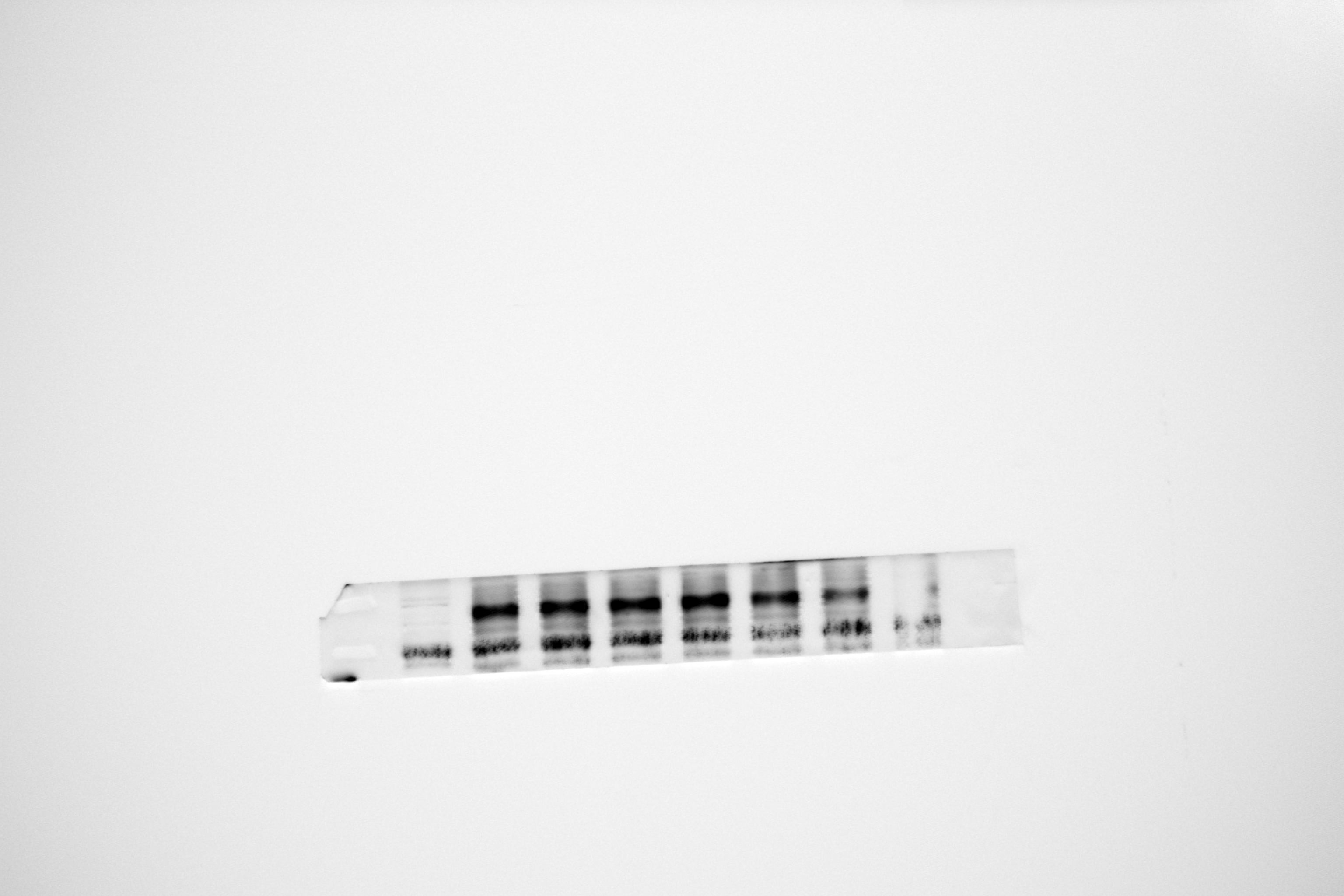

Supplement: Figure 1—figure supplement 1—source data 1. [file elife-106934-fig1-figsupp1-data1.zip › Figure 1 - figure supplement 1 - source data 1/Fig S1D BBMEF Flag ab.tif]

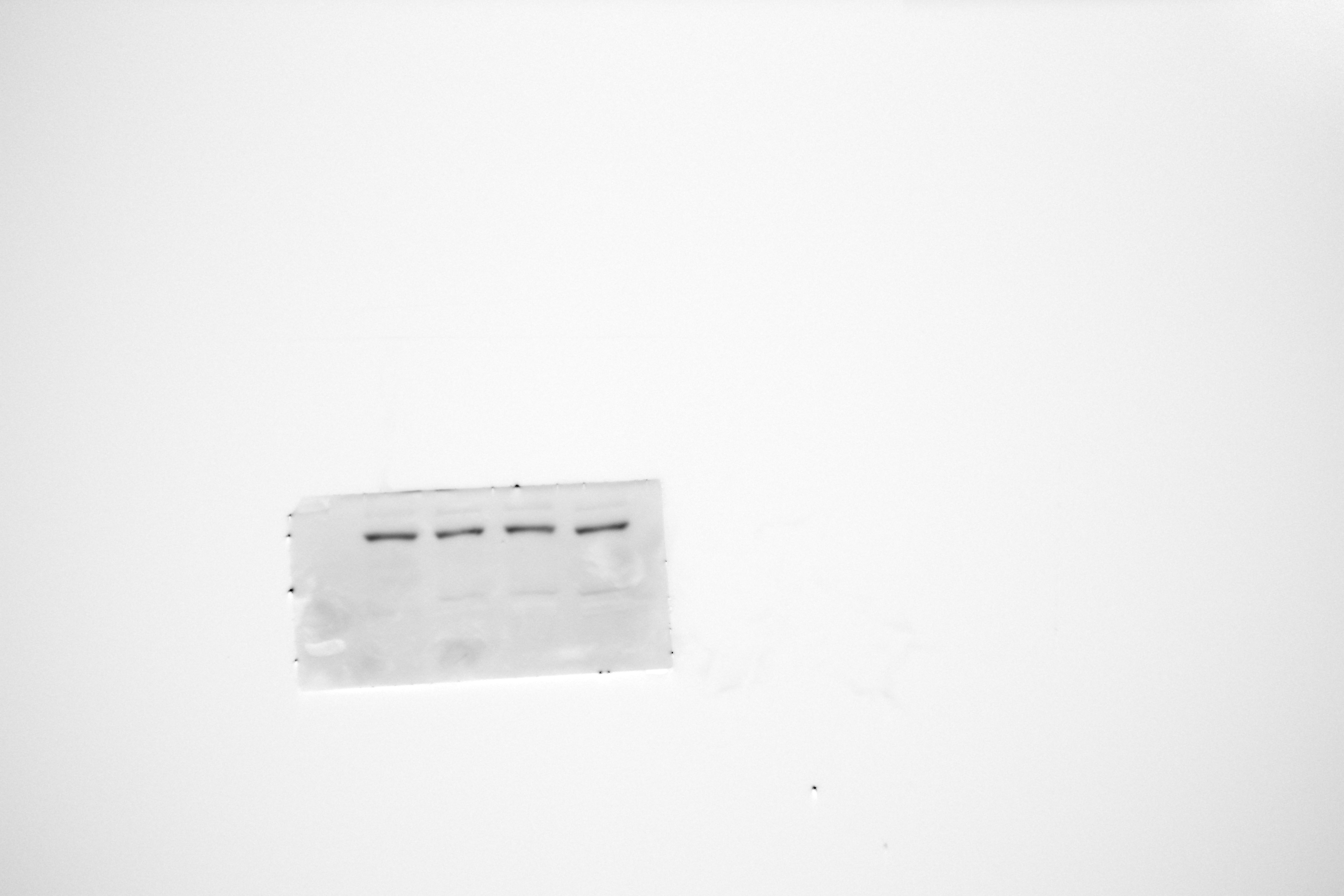

Supplement: Figure 1—figure supplement 1—source data 1. [file elife-106934-fig1-figsupp1-data1.zip › Figure 1 - figure supplement 1 - source data 1/Fig S1D BBMEF LaminB1 ab.tif]

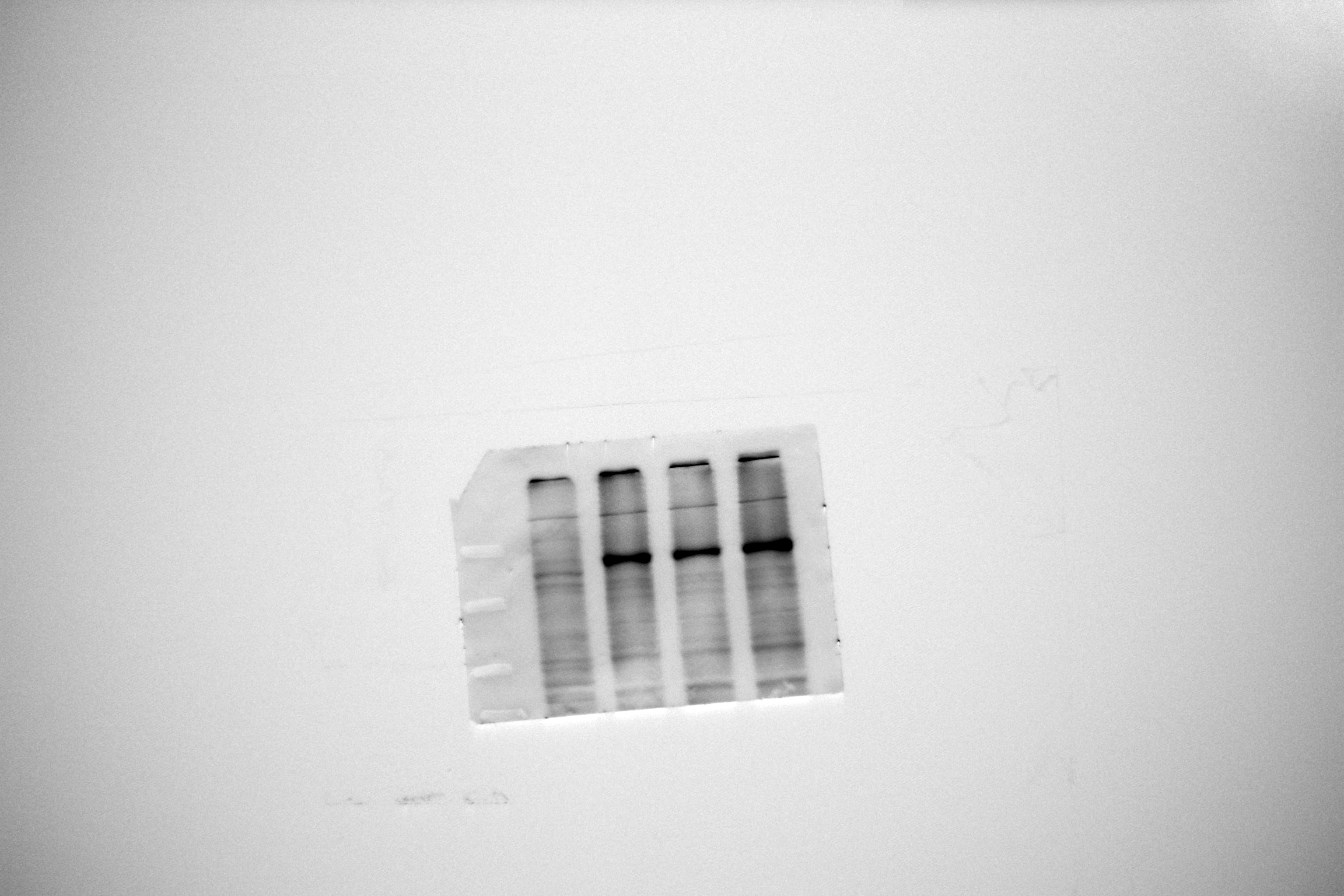

Supplement: Figure 1—figure supplement 1—source data 1. [file elife-106934-fig1-figsupp1-data1.zip › Figure 1 - figure supplement 1 - source data 1/Fig S1D BBMEF myc ab.tif]

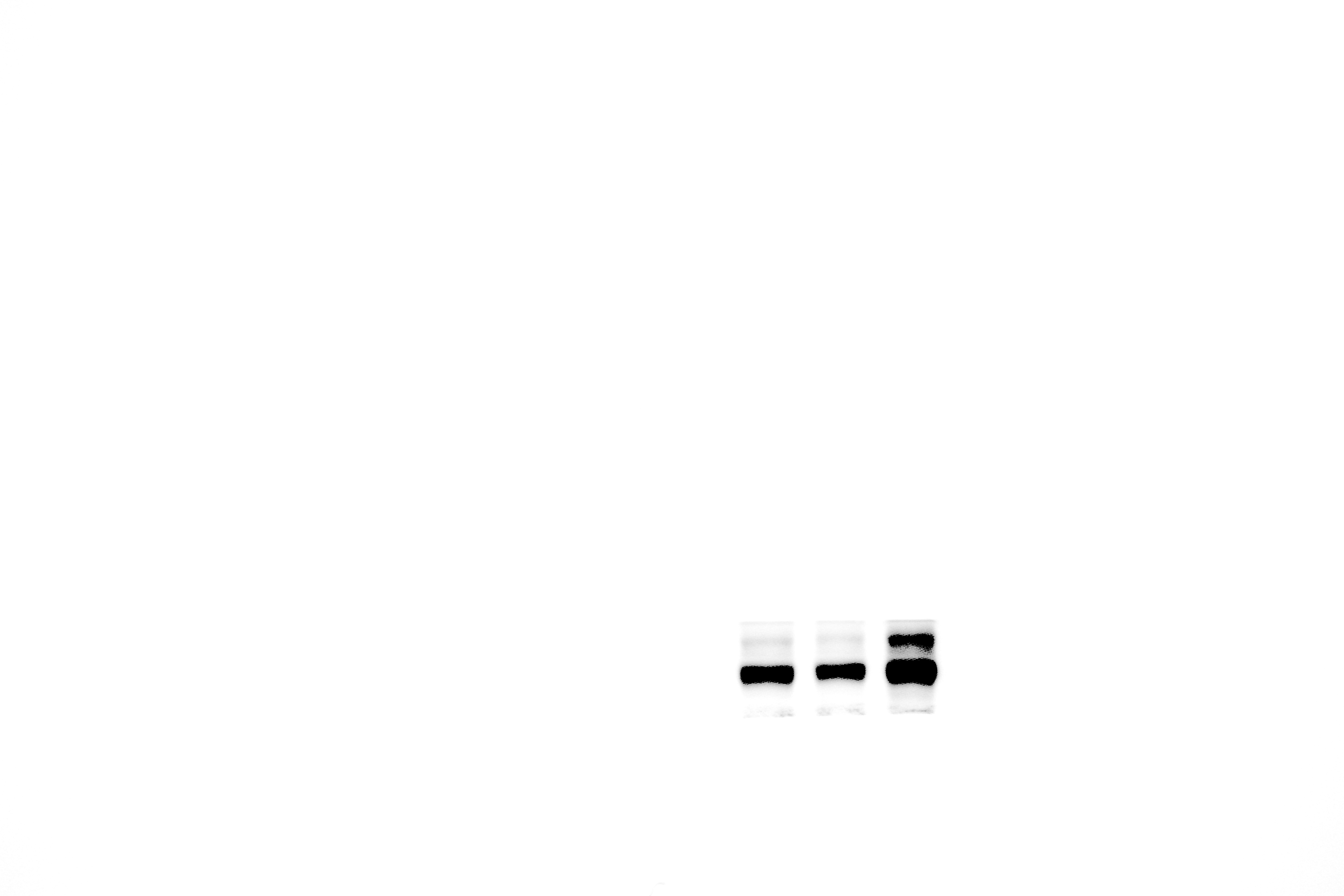

Supplement: Figure 1—figure supplement 1—source data 1. [file elife-106934-fig1-figsupp1-data1.zip › Figure 1 - figure supplement 1 - source data 1/Fig S1D WRNHF Flag ab.tif]

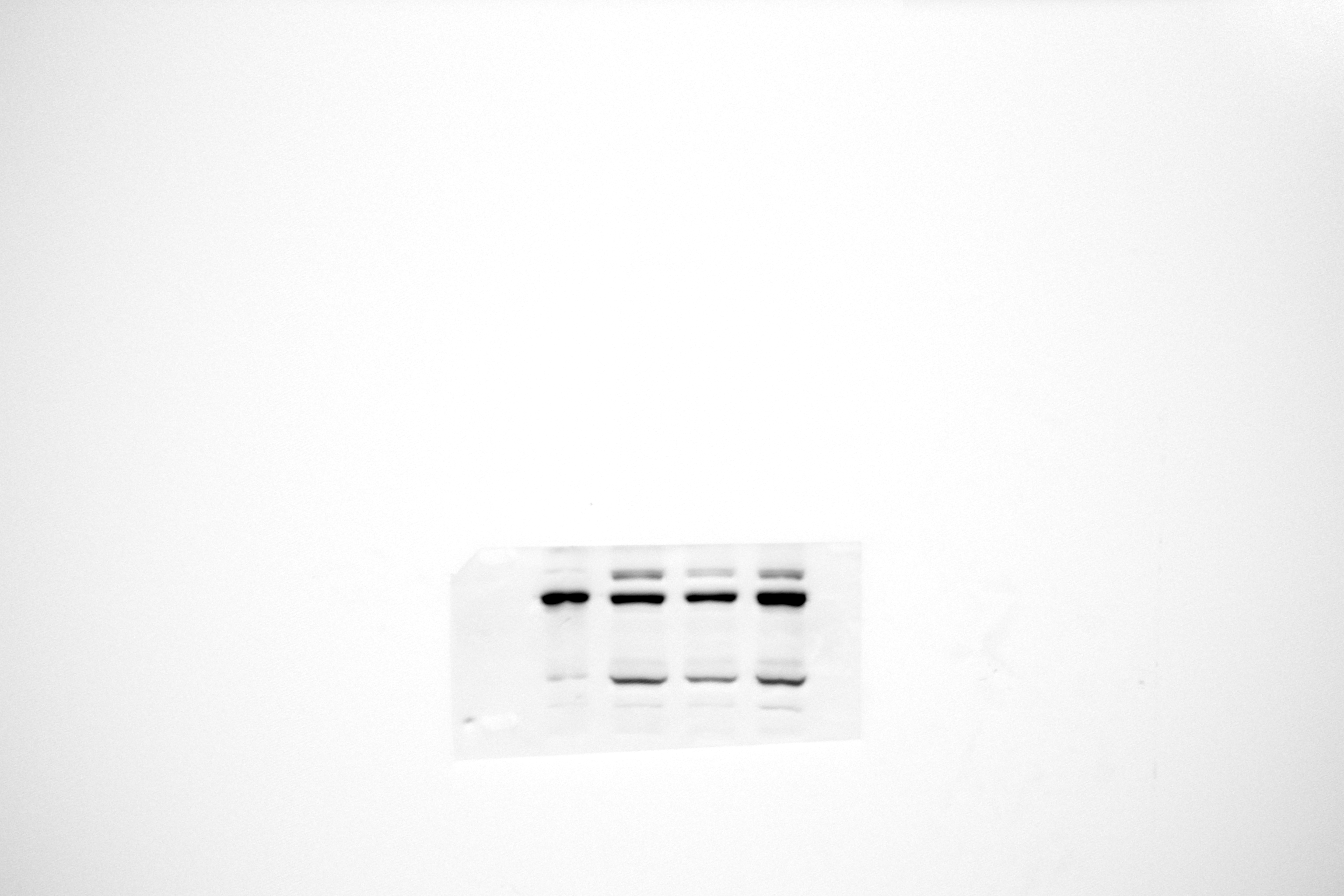

Supplement: Figure 1—figure supplement 1—source data 1. [file elife-106934-fig1-figsupp1-data1.zip › Figure 1 - figure supplement 1 - source data 1/Fig S1D WRNHF LaminB1 ab.tif]

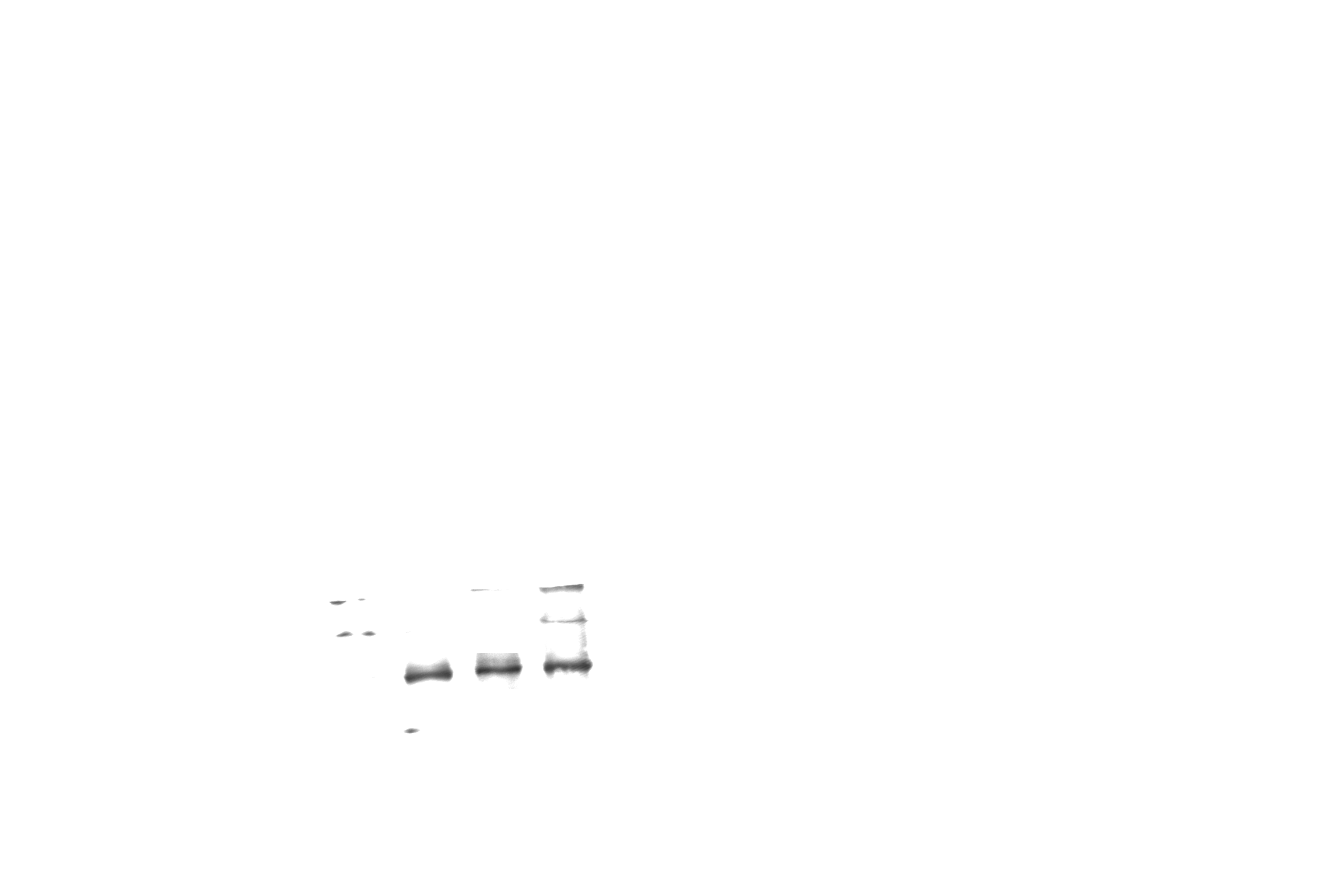

Supplement: Figure 1—figure supplement 1—source data 1. [file elife-106934-fig1-figsupp1-data1.zip › Figure 1 - figure supplement 1 - source data 1/Fig S1D WRNHF myc ab.tif]

Figure 1 -figure supplement 1 - source data 2 (A,B)

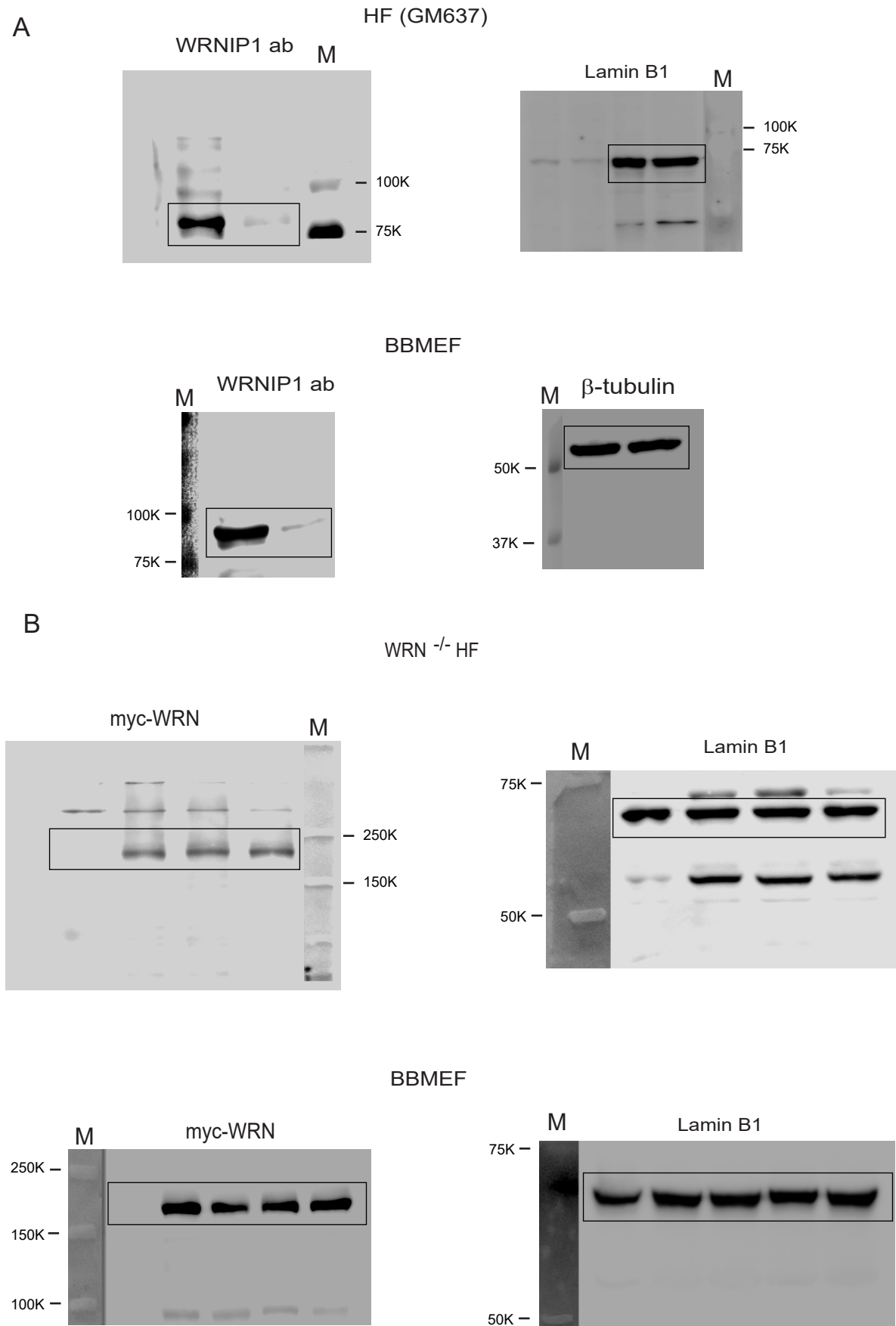

Figure 1 - figure supplement 1 - source data 2 (C,D)

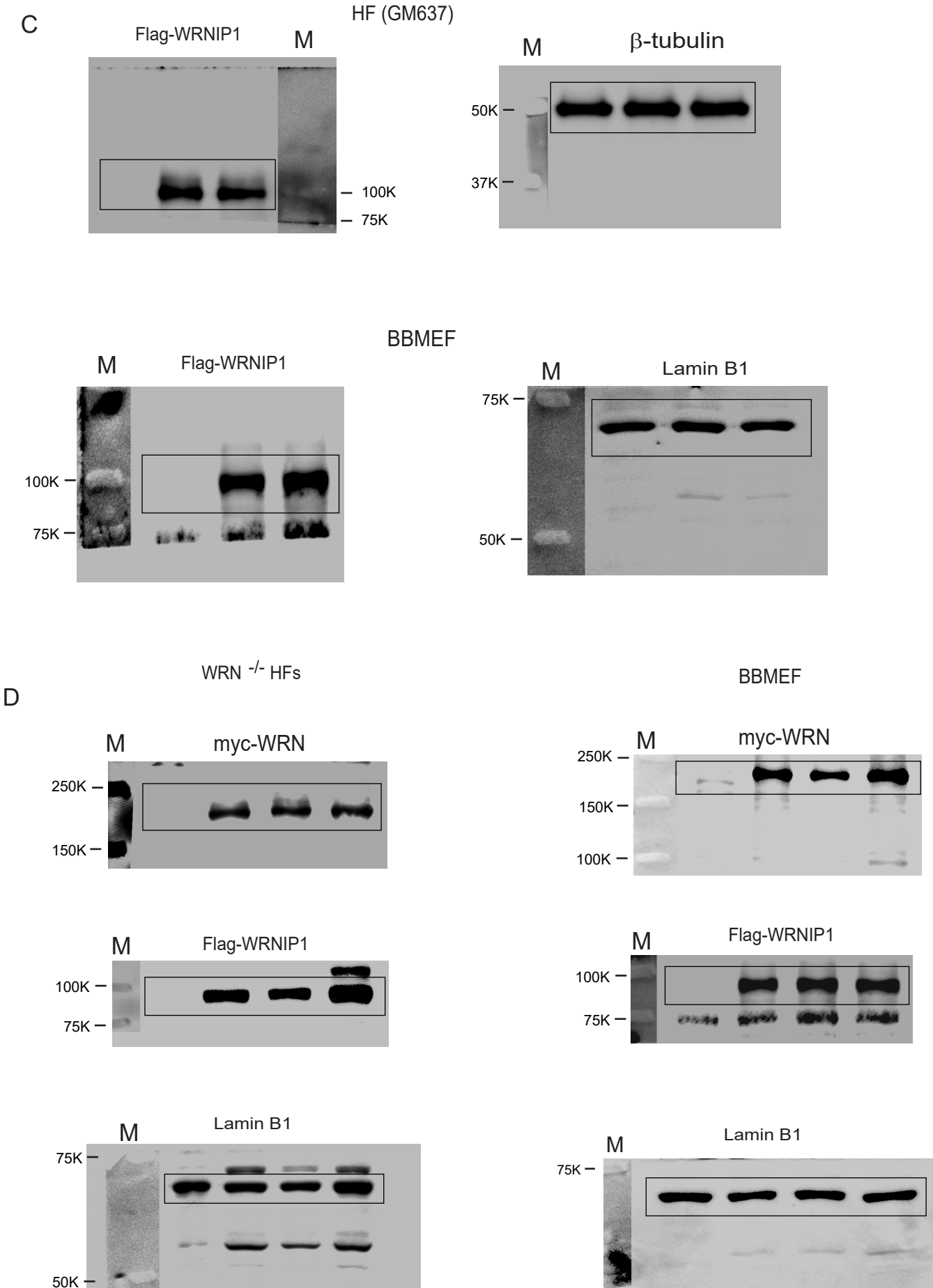

Supplement: Figure 1—figure supplement 1—source data 2. [file elife-106934-fig1-figsupp1-data2.pdf]
