# Supplementary material for: WRN and WRNIP1 ATPases impose high fidelity on translesion synthesis by Y-family DNA polymerases
Source: eLife. 2025 Sep 3;14:RP106934. doi: 10.7554/eLife.106934 (PMC12408069; doi:10.7554/eLife.106934)
Supplement: Table 1—source data 1. [file elife-106934-table1-data1.pdf]

Table 1 Source data

siRNA	Vector expressing:	
WRN	Myc-WT-WRN	(no UV)
	18.4	
	17.6	
	15.7	
WRN	Myc-WT-WRN	(+UV)
	47.8	
	42.4	
	50.6	
WRN	Myc-E84A-WRN	(+UV)
	68.3	
	78.6	
	74.2	
WRN	Myc-K577A-WRN	(+UV)
	75.6	
	78.5	
	80.4	
WRN	Myc-E84A,K577A-WRN	(+UV)
	108.7	
	98.6	
	102.6	
WRNIP1	Flag-WT-WRNIP1	(+UV)
	45.3	
	44.2	
	48.4	
WRNIP1	Flag-K274A-WRNIP1	(+UV)
	86.8	
	77.9	
	73.7	
	79.3	
WRN + WRNIP1	Myc-E84A-WRN + Flag-K274A-WRNIP1	(+UV)
	94.2	
	111.6	
	102.8	
WRN + WRNIP1	Myc-K577A-WRN + Flag-K274A-WRNIP1	(+UV)
	102.6	

	106.6	
	106.9	
	108.2	
WRN + WRNIP1	Myc-E84A,K577A-WRN + Flag-K274A-WRNIP1	(+UV)
	136.4	
	129.2	
	140.6	
	138.8	
WRN + Polθ	Myc-WT-WRN	(+UV)
	22.1	
	19.4	
	18.2	
WRN + Polθ	Myc-E84A-WRN	(+UV)
	49.8	
	50.2	
	52.1	
	46.8	
WRN + Polθ	Myc-K577A-WRN	(+UV)
	56.8	
	54.7	
	51.4	
WRN + Polθ	Myc-E84A,K577A-WRN	(+UV)
	81	
	76	
	73.6	
	82.4	
WRNIP1 + Polθ	Flag-WT-WRNIP1	(+UV)
	23.1	
	20.4	
	18.2	
WRNIP1 + Polθ	Flag-K277A-WRNIP1	(+UV)
	62.4	
	54.7	
	63.2	
ART558 (20μM)		(+UV)
	19.9	
	21.2	
	20.8	

WRN + WRNIP1/ART558 (20μM)	Myc-E84A-WRN + Flag-K274A-WRNIP1	(+UV)
	78.3	
	76	
	77.3	
WRN + WRNIP1/ART558 (20μM)	Myc-K577A-WRN + Flag-K274A-WRNIP1	(+UV)
	80.5	
	77.3	
	78.8	
