# Supplementary material for: WRN and WRNIP1 ATPases impose high fidelity on translesion synthesis by Y-family DNA polymerases
Source: eLife. 2025 Sep 3;14:RP106934. doi: 10.7554/eLife.106934 (PMC12408069; doi:10.7554/eLife.106934)
Supplement: Figure 4—source data 1. [file elife-106934-fig4-data1.pdf]

Fiigure 4A Source data

siRNA	Vector expressing	
WRN	Myc-WT-WRN	(no UV)
	11.9	
	15.4	
	16.4	
WRN	Myc-WT-WRN	(UV)
	27.3	
	29.4	
	28.3	
WRN	Myc-E84A-WRN	(UV)
	48.2	
	44.7	
	45.6	
WRN	Myc-K577A-WRNA	(UV)
	42.6	
	50.2	
	48.8	
WRN	Myc-E84A,K577A-WRN	(UV)
	72	
	69.6	
	72.4	
WRNIP1	Flag-WT-WRNIP1	(UV)
	30.9	
	27.4	
	30.2	
WRNIP1	Flag-K274A-WRNIP1	(UV)
	50.7	
	48.8	
	52.6	
WRN + WRNIP1	Myc-E84A-WRN + Flag-K274A-WRNIP1	(UV)
	65.8	
	71.3	
	74.4	
	70.2	
WRN + WRNIP1	Myc-K577A-WRN + Flag-K274A-WRNIP1	(UV)

	70.6	
	72.4	
	68.6	
	70.8	
WRN + WRNIP1	Myc-E84A, K577AWRN + Flag-K274A-WRNIP1	(UV)
	89.6	
	91.4	
	90.7	
