# Supplementary material for: WRN and WRNIP1 ATPases impose high fidelity on translesion synthesis by Y-family DNA polymerases
Source: eLife. 2025 Sep 3;14:RP106934. doi: 10.7554/eLife.106934 (PMC12408069; doi:10.7554/eLife.106934)
Supplement: Supplementary file 1. [file elife-106934-supp1.docx]

**Supplementary file 1A. TLS frequencies opposite a Tg lesion carried on the leading strand DNA template of a duplex plasmid in WT HFs or WRN^-/-^ HFs expressing WRN and/or WRNIP1 mutant proteins**

| HFs | siRNA | Vector expressing | # *Kan^+^* colonies | # blue colonies among *Kan^+^* | TLS (%) |
| --- | --- | --- | --- | --- | --- |
| WT | NC | - | 374 | 84 | 22.5 |
|  | WRNIP1 | Vector control | 338 | 37 | 10.9 |
|  | WRNIP1 | WT-WRNIP1 | 331 | 70 | 21.1 |
|  | WRNIP1 | K274A-WRNIP1 | 340 | 74 | 21.8 |
| WRN ^-/-^ | NC | Vector control | 311 | 36 | 11.6 |
|  | NC | WT-WRN | 403 | 95 | 23.6 |
|  | NC | E84A-WRN | 344 | 74 | 21.5 |
|  | NC | K577A-WRN | 294 | 62 | 21.1 |
|  | NC | E84A,K577A-WRN | 307 | 68 | 22.1 |
|  | WRNIP1 | K577A-WRN +  K274A-WRNIP1 | 327 | 70 | 21.4 |
|  | WRNIP1 | E84A,K577A-WRN +  K274A-WRNIP1 | 335 | 72 | 21.5 |

**Supplementary file 1B. TLS frequencies opposite an εdA lesion carried on the leading strand DNA template of a duplex plasmid in WT HFs or WRN^-/-^ HFs expressing WRN and/or WRNIP1 mutant proteins**

| HFs | siRNA | Vector expressing | # *Kan^+^* colonies | # blue colonies among *Kan^+^* | TLS (%) |
| --- | --- | --- | --- | --- | --- |
| WT | NC | - | 408 | 92 | 22.5 |
|  | WRNIP1 | Vector control | 294 | 24 | 8.2 |
|  | WRNIP1 | WT-WRNIP1 | 254 | 58 | 22.8 |
|  | WRNIP1 | K274A-WRNIP1 | 311 | 72 | 23.2 |
| WRN^-/-^ | NC | Vector control | 348 | 32 | 9.2 |
|  | NC | WT-WRN | 208 | 38 | 18.3 |
|  | NC | K577A-WRN | 218 | 42 | 19.3 |
|  | NC | E84A,K577A-WRN | 236 | 45 | 19.1 |
|  | WRNIP1 | E84A-WRN+  K274A-WRNIP1 | 304 | 65 | 21.4 |
|  | WRNIP1 | E84A,K577A-WRN +  K274A-WRNIP1 | 288 | 60 | 20.8 |
|  | WRNIP1 | K577A-WRN +  K274A-WRNIP1 | 302 | 64 | 21.2 |
|  | WRNIP1+Polι | E84A,K577A -WRN +  K274A-WRNIP1 | 252 | 26 | 10.3 |
